# Poly(Ionic Liquid) Nanoparticles Selectively Disrupt Biomembranes

**DOI:** 10.1002/advs.201801602

**Published:** 2018-12-17

**Authors:** Eleanor Ewins, Rafael B. Lira, Weiyi Zhang, Jiayin Yuan, Markus Antonietti, Tom Robinson, Rumiana Dimova

**Affiliations:** ^1^ Department of Theory & Bio‐Systems Max Planck Institute of Colloids and Interfaces Science Park Golm 14424 Potsdam Germany; ^2^ Department of Colloid Chemistry Max Planck Institute of Colloids and Interfaces Science Park Golm 14424 Potsdam Germany

**Keywords:** antifungal, antimicrobial activity, giant vesicles, membrane permeabilization, microfluidics, pores

## Abstract

Polymer‐based nanoparticles have an increasing presence in research due to their attractive properties, such as flexible surface functionality design and the ability to scale up production. Poly(ionic liquid) (PIL) nanoparticles of size below 50 nm are very unique in terms of their high charge density and internal onion‐like morphology. The interaction between PIL nanoparticles and giant unilamellar vesicles (GUVs) of various surface charge densities is investigated. GUVs represent a convenient model system as they mimic the size and curvature of plasma membranes, while simultaneously offering direct visualization of the membrane response under the microscope. Incubating PIL nanoparticles with GUVs results in poration of the lipid membrane in a concentration‐ and charge‐dependent manner. A critical poration concentration of PILs is located and the interactions are found to be analogous to those of antimicrobial peptides. Microbial mimetic membranes are already affected at submicromolar PIL concentrations where contrast loss is observed due to sugar exchange across the membrane, while at high concentrations the collapse of vesicles is observed. Finally, a confocal microscopy–based approach assessing the particle permeation through the membrane is reported and a mechanism based on bilayer frustration and pore stabilization via particle integration in the membrane is proposed.

## 1 Introduction

The study of nanoparticle interactions with biomembranes is an increasingly relevant research field because of applications in medical imaging,^1^ drug delivery, and antibacterial activity.^2^ Such interactions with cell membranes are complex, due to the dynamic nature of the membrane and the large range of constituting components,^3^ and thus it presents a challenge to fully understand every step within the interaction pathway. Additional complexity is introduced by cell metabolism and growth.^4^ To overcome this hurdle, model membrane systems are employed, as they mimic the types of (toxic) interactions evoked upon contact with nanomaterials.^5^ When evaluating the effects that particles and other membrane‐active agents have on membrane properties and integrity, such as changes in permeability, bulk assays involving leakage from spherical large or small unilamellar vesicles (LUVs and SUVs, respectively, of size ≈20–100 nm) are typically employed.^6^ However, working with vesicles of such small sizes can raise questions regarding the role that membrane curvature can play. Their size also means that they cannot be directly imaged, thus measurements are indirect, require more advanced analysis, and the conclusions about the vesicle integrity are futile as direct kinetic observations of the membrane are not feasible. An alternative model system is provided by giant unilamellar vesicles (GUVs).^7^ Having sizes in the range 10–100 µm, they offer the possibility of directly imaging the response of the membrane and the vesicle stability as well as characterizing and visualizing the interaction of particles with the membrane under the microscope. These advantages have been utilized in other particle–membrane studies for both micro‐ and nanoparticles, where phenomena such as particle wrapping and membrane deformation and poration have been observed.^8^

Here, we used GUVs to examine the effects that a new/emergent class of nanoparticles made of poly(ionic liquid) (PIL) have on biomembranes. The PIL nanoparticles used in this work were below 50 nm in size and were formed via dispersion polymerization of vinylimidazolium‐type ionic liquid monomers.^9^ These particles combine the attractive properties of polymers, such as flexible functionality, with the additional properties afforded by ionic liquids.^10^ PILs have been used already in a multitude of applications, covering aspects from interface mediation and adhesion, to sensor devices, battery membranes, or organic photovoltaic cells. In the present context, it is important that, in spite of their often polycationic character, they seem to be rather nontoxic to higher animals and cells, and the activity of many enzymes is preserved in ionic liquid and PIL environments. PILs derived from alkylvinylimidazolium salts with long alkyl substituents assemble into multilamellar onion‐like structures of concentric layers. Ionic liquids themselves have been shown to have significant antimicrobial properties^11^ and also to act against bacteria, fungi, and algae.^12^ When in polymeric form ionic liquids still act against microbes, as demonstrated by the antibacterial properties of PIL brushes.^13^ In the context of model membrane systems, and in particular GUVs, the activity of an antimicrobial agent, typically an antimicrobial peptide, can be observed via leakage of molecules into or out of GUVs,^14^ changes in membrane morphology (thickening of the membrane),^15^ or GUV bursting.^16^ Considering the unique combination of the properties of PIL coupled with the membrane‐active nature of nanoparticles, and their potential use as an antimicrobial agent, we explore the interaction of PIL nanoparticles with giant vesicles. The GUVs were composed of neutral or negatively charged lipids to distinguish the effect of PILs on membranes with mammalian vs bacterial mimetics. Positively charged membranes were also explored with the aim of probing the electrostatic dependence of the interactions. It has been previously shown that charged membranes are required for the action of antimicrobial molecules.^17^ We determined the lytic activity of the PIL nanoparticles and observed their disruptive effect on membranes using microfluidic chambers. We also synthesized fluorescently labeled particles to determine the binding and translocation of PILs across the membrane. The changes in overall membrane properties were assessed from the morphological appearance of the vesicles and molecular rearrangement was monitored from diffusion measurements.

## 2 Results

### 2.1 PILs Induce Vesicle Leakage

**Figure 1:**
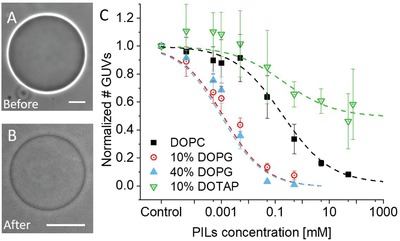
Permeation and bursting of GUVs in the presence of PILs. A,B) GUVs composed of DOPC/DOPG 90/10 mol% (10% DOPG) viewed before and after incubation with 5 × 10^−6^ m PILs showing loss of optical contrast resulting from the particle–membrane interactions. Scale bars: 10 µm. C) Plot of the number of surviving vesicles after incubation with PIL solutions normalized by the number of vesicles in the absence of particles. The data for the different membrane compositions are averaged from three independent preparations (raw data can be found in Figure S4 in the Supporting Information). The curves represent sigmoidal fits in Origin.

The PILs have sizes of 24.0 ± 6.5 nm in a dried state and 27.8 ± 10.1 nm in a dispersion state, and positive surface charge of 45.1 ± 0.9 mV as assessed with transmission electron microscopy, dynamic light scattering, and electrophoretic mobility measurements, respectively (see the Experimental Section and Figure S1 in the Supporting Information). The GUVs were prepared to contain different molar fractions of the neutral lipid 1,2‐dioleoyl‐*sn*‐glycero‐3‐phosphocholine (DOPC) and the negatively charged 1,2‐dioleoyl‐*sn*‐glycero‐3‐[phospho‐*rac*‐(1‐glycerol)] (DOPG), with the aim of mimicking the composition of mammalian and bacterial membranes, respectively. The nanoparticles were initially incubated for 1 h with GUVs composed of DOPC/DOPG in a 90/10 molar ratio, providing an overall negative charge to the membrane. Sugar asymmetry across the GUV membrane was used to aid visualization due to the difference in refractive indices of sucrose and glucose when viewed in phase contrast mode (**Figure** **1**A). Upon the addition of 5 × 10^−6^ m PILs, an exchange of sugars across the vesicle membrane occurs, as evidenced by loss in optical contrast in Figure 1B. Sugar molecule exchange signifies formation of nanometer pores in the vesicle membrane. In addition, the number of surviving vesicles after incubation with PILs was found to decrease suggesting that some of the vesicles have burst.

To further evaluate the effect of PILs on GUVs, a range of PIL concentrations were incubated with vesicles of four different lipid compositions: pure DOPC, DOPC/DOPG 90/10 mol% (10% DOPG), DOPC/DOPG 60/40 mol% (40% DOPG), and DOPC/1,2‐dioleoyl‐3‐trimethylammoniumpropane (chloride salt) (DOTAP) 90/10 mol% (10% DOTAP). Of these lipids, DOPC is neutral, DOPG is negatively charged, and DOTAP is positively charged (see Figure S2 in the Supporting Information for the lipid structures). Nanoparticle concentrations between 0.5 × 10^−6^ and 90 × 10^−3^ m (total PIL monomer concentration) were explored. Positively charged particles can cause adhesion of the GUVs to the negatively charged glass walls of the observation chamber, followed by vesicle rupture. Conventionally employed glass coating with bovine serum albumin (BSA) and casein was not found to avoid this. We considered using solutions of higher salinity to potentially screen the interactions with the glass, but this would have presumably changed the interactions with the membrane as well. Furthermore, preparing vesicles in high‐salinity solutions for the explored membrane compositions is not straightforward^18^ and is associated with certain difficulties considering the membrane compositions we explored. The alternative of preparing the vesicles in sugar solution and diluting them in salt solutions was also discarded because of effects associated with phase state^19^ and membrane tubulation resulting from spontaneous curvature generation.^20^ Thus, before observation, the vesicles were immobilized in agarose (0.2% by weight)^21^ as described in the Experimental Section. From stacks of images in the *z*‐direction, we could examine the number of surviving vesicles for a fixed sample volume. The number of surviving GUVs normalized by the number of control vesicles (no PILs added) in the same volume was plotted as a function of particle concentration (Figure 1C). Vesicles were counted for all sizes above 4 µm and excluded if they had pronounced defects (see Figure S3 in the Supporting Information for examples of included and excluded vesicles). Note that the yield of vesicles prepared from different lipid mixtures varied significantly. The raw, averaged vesicle population data can be found in Figure S4 in the Supporting Information. With increasing PIL concentrations, all lipid compositions experience a decrease in population size. Notably, the onset of vesicle loss has a dependence on membrane charge, with negatively charged GUVs seeing a decrease in population at a lower PIL concentration than neutral and positively charged GUVs.

The poration and rupture of the vesicles by the PILs could be due to increased tension as the membrane bends and wraps around the particles. This increased tension can then be relieved by the formation of pores in the membrane and a reduction in the internal volume. Short‐lived micrometer‐sized pores (with lifetimes in the 10–100 ms range as observed in phosphatidylcholine (PC) membranes upon electroporation^22^) are less likely to be the cause of loss of contrast because they reseal rapidly due to the high membrane edge tension,^23^ leaving the vesicle contrast preserved. We conclude that rather the membrane develops small and stable pores, which allow the full exchange of the internal and external solutions in tens of seconds as observed (see Figure S5 in the Supporting Information). The pores appear to be stable for hours judging from the lack of vesicle deformation when exposed to electric fields as those applied in ref. 23 (data not shown), which is an indication of a permeable membrane.

A similar poration effect (observed via a loss of contrast) is also reported for the action of antimicrobial peptides on GUVs.[[qv: 17a]] As such, we assessed the vesicles' responses to the PILs in a comparable manner, by determining the minimum bursting concentration (MBC) of the PILs.^16^ The MBC is defined as the minimum concentration required to induce extensive GUV bursting (>90%). The values attained for the MBC of GUVs can be directly compared with the minimal inhibitory concentration on microorganisms as was done previously for antimicrobial peptides.^16^, ^24^ Thus, the lytic activity on GUVs can be used to predict how antimicrobial agents work in vivo. From the data in Figure 1C, we determine the MBC of PILs for each membrane composition (see **Table** **1**).

**Table 1:** The MBC values of PILs for membranes of different lipid compositions. The MBC (i.e., PIL concentration required to reduce the vesicle population to 10%) has a strong dependence on membrane charge. An MBC could not be determined for 10% DOTAP membranes as the population does not reach 10% survival within experimental limits

Membrane composition	MBC [×10^−3^ m]	Error (standard deviation)
40% DOPG	0.03	0.01
10% DOPG	0.19	0.10
Pure DOPC	42	14
10% DOTAP	>90	–

There is no distinctive difference in the response of the two negatively charged GUV populations as they both reach the MBC 10% survival population within a concentration range of (0.03–0.2) × 10^−3^ m. At the MBC values for the negatively charged membranes, over 60% of the DOPC population survives. The neutral membranes reach the MBC at (42 ± 14) × 10^−3^ m. The positively charged GUVs (10% DOTAP) show the most resistance to the positively charged PILs, with the population never falling below 30% for the maximum PIL concentration reached. It should be noted that the large error bars in Figure 1C are due to a smaller population size for this lipid composition—DOTAP‐doped membranes not only are notoriously more difficult to form^25^ but also are not found in nature. The use of this lipid composition helps to probe the contribution that membrane composition has on the PIL–membrane interaction. It is clear that there is a strong dependence on charge, and that the electrostatic interactions govern the final outcome.

The negative vesicles, representing bacterial mimetics, reach the 10% survival population at a concentration several orders of magnitude lower than that of the neutral vesicles (mammalian mimetics). This is of high biological significance if these particles are to be utilized as an antibacterial agent, as most bacterial membranes have an overall negative surface charge.^26^ Due to the significant affinity for negative membranes that these particles exhibit, we believe that the charge‐mediated interaction between the two is the source of the initial adhesion of the particles to the membrane.

The results in Figure 1C show the number of surviving vesicles but not their state. Considering that nanoparticle incubation induced leakage and extensive morphological changes on the vesicles (see below), we investigated the change in size of the GUVs. All compositions were found to exhibit a decrease in the average vesicle size as a function of PIL concentration, as shown in Figure S6 in the Supporting Information. However, the change in size (normalized to the average size for each composition at 0 m PILs) appears to have only a weak dependence on charge (Figure S6, Supporting Information). The negatively charged populations, in most instances, have smaller average sizes than DOPC and 10% DOTAP membranes.

### 2.2 Dynamics of GUV Response

**Figure 2:**
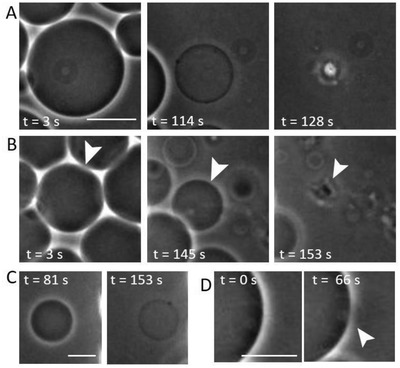
Dynamics of morphological changes of 10% DOPG GUVs when exposed to 0.1 × 10^−3^ m PILs in a microfluidic chamber (the nanoparticles are introduced from the right). Three types of vesicle response are presented. A) Vesicle decreases in size, becomes permeable, and bursts. B) Vesicle decreases in size, retains contrast, and bursts. C) Vesicle decreases slightly in size, loses contrast, but remains intact. D) (Macro)pore formation in a vesicle with preserved contrast, as visualized by the changes in the bright halo around the vesicle interrupted in the area of the pore. Sequences taken from Movie S1 in the Supporting Information. Scale bars: 15 µm.

To deal with the disadvantage of bulk assays (as those applied above) where the vesicle history during the incubation stage remains unknown, we used a microfluidic device to observe the interactions between the PILs and the GUVs directly as they occurred (see the Experimental Section). In addition to being able to observe the same GUV, the setup also allows tracking of the interactions as complete fluid exchange is performed. The microfluidic device was filled with 10% DOPG vesicles, as negative GUVs have been found to be more affected, and a solution of PILs at 0.1 × 10^−3^ m was introduced (see Movie S1 in the Supporting Information). A significant number of GUVs are destroyed. The particles may mediate adhesion of the vesicles to the glass leading to tension increase and rupture. Throughout these interactions, the vesicles typically undergo one of three interaction pathways (**Figure** **2**): a loss of phase contrast with simultaneous reduction in vesicle diameter followed by vesicle destruction (Figure 2A); preserved contrast, a reduction in vesicle size, and vesicle destruction (Figure 2B); and a loss of contrast, vesicle size preservation, and the vesicle survives throughout observation (Figure 2C). Instances of (macro)pore formation, the cause of membrane leakage, were also observed (Figure 2D). The different response pathways could be due to i) the individual vesicle properties, as the preparation protocol offers limited control over lipid composition at the individual vesicle level,^27^ or ii) different surface properties of the chamber as some vesicles may have ruptured and spread, thus preventing others from bursting due to particle‐mediated adhesion to the glass.

Another factor determining the type of vesicle response is the initial membrane tension that can significantly vary within the population after preparation and has values in the range between 10^−6^ and 1 mN m^−1^.^28^ Thus, vesicles having more excess area (low tension) could potentially wrap the particles while tense ones might already reach the lysis tension of ≈5–10 mN m^−1^ 29 after coming into contact with only a few particles and then burst.

The vesicle‐to‐vesicle variation of losing or retaining contrast suggests different types of pores. For vesicle size to decrease, such as in Figure 2A,B, the internal volume must decrease, most likely through the expulsion of the internal sucrose solution. In Figure 2A, there is not only a size decrease but also a loss of contrast suggesting that also influx of glucose is established as the external and internal solutions become the same. This could suggest the formation of more stable/long‐lived pores to allow full mixing of the solutions across the membrane. In the microfluidic device, we could not avoid the contact of the vesicles with the substrate, which may additionally contribute to increasing the tension due to adhesion.

### 2.3 Membrane Coverage by PILs

After observing how the particles affect the vesicle populations, we attempted to characterize the surface concentration of PIL on the vesicles. To do so, the PILs were fluorescently labeled with rhodamine B (Rh‐PILs) (see the Experimental Section). To ensure that the labeling process and the presence of the dye in the particle structure did not change how the PILs interacted with the GUVs, we repeated the statistical experiments for the number of GUVs at various concentrations of Rh‐PILs (see Figure S7 in the Supporting Information for comparison between the labeled and nonlabeled PILs). The number of surviving vesicles exhibits the same trend with increasing PIL concentration. As such, we can assume that the presence of the dye does not impinge on the interaction of the PILs with the membranes.

**Figure 3:**
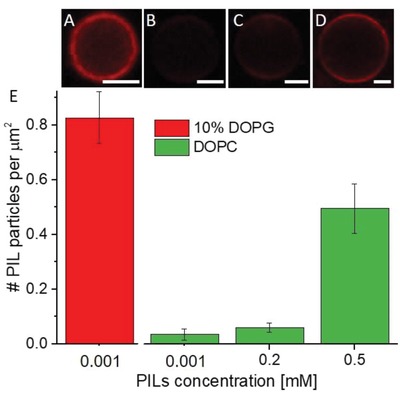
Fluorescently labeled PILs on unlabeled GUV membranes of different composition and area occupied per PIL particle as calculated from such images: A) 0.001 × 10^−3^ m Rh‐PILs on 10% DOPG; B) 0.001 × 10^−3^ m Rh‐PILs on DOPC; C) 0.2 × 10^−3^ m Rh‐PILs on DOPC; and D) 0.5 × 10^−3^ m Rh‐PILs on DOPC. Scale bars: 5 µm. E) Number of PILs per 1 µm^2^ vesicle area. The error bars represent standard errors assessed from nine to ten vesicles per composition. See also Figure S8 in the Supporting Information for raw intensity data.

Confocal microscopy observation of the incubated GUVs with Rh‐PILs showed that the particles were in fact present on the membrane and that a percentage had crossed to the GUV interior (see below). **Figure** **3**A–D shows the fluorescence from nonlabeled membranes after 1 h incubation with Rh‐PILs. The fluorescent signal demonstrates that the PILs enrich on the membrane of the GUVs (at concentrations that cause leakage and size decrease of the vesicles, but do not destroy all vesicles in the sample). This signal comes purely from rhodamine B (Rh‐B) in the PIL sample, as the GUVs were prepared in the absence of any dye; we obtained control images of the bare membranes that show there is no/background fluorescence (Figure S9, Supporting Information). Considering first the intensity values from the PILs at the membrane, we already observe a difference between vesicles with different lipid compositions (Figure 3). The membrane intensities were assessed by measuring the average pixel intensity for a fixed section of the membrane (see Figure S10 in the Supporting Information), taking care to account for any dye polarization effects. For the same concentration of PILs (0.001 × 10^−3^ m), the membrane intensity for the 10% DOPG membrane (21.23 ± 12.75 AU) is tenfold higher than that of DOPC membranes (2.82 ± 1.81 AU). When we then increase the concentration of PILs to 0.5 × 10^−3^ m (a concentration at which approximately 50% of the DOPC population has been destroyed, as is also the case for the 10% DOPG vesicles at 0.001 × 10^−3^ m), the average membrane intensity increases (13.71 ± 6.44 AU) but is still notably lower than for the 10% DOPG membranes with a much lower concentration of PILs (0.001 × 10^−3^ m). The intensity values are of course related to the number of particles on the membrane, which we now go on to quantify.

In order to determine the number of particles on the membrane from the Rh‐PIL fluorescence intensity measured on the GUVs, we first assessed the efficiency of the particle labeling with Rh‐B. We dissolved the particles in ethanol and deduced the Rh‐B concentration from the extinction coefficient of the system. The concentration of Rh‐B in 14.96 mg mL^−1^ PILs was found to be 9.1 × 10^−4^ mg mL^−1^ and the corresponding number of Rh‐B molecules per PIL particle was roughly assessed to be 2.3 ± 2 (see Section S1 in the Supporting Information). Then, to convert the fluorescence intensity from the images (as in Figure 3A–D) to Rh‐B concentration in the membrane, we constructed a calibration curve of the fluorescence intensity of vesicles as a function of the concentration of a lipid labeled with a similar fluorophore, 1,2‐dipalmitoyl‐*sn*‐glycero‐3‐phosphoethanolamine‐*N*‐(lissamine rhodamine B sulfonyl) ammonium salt (Rh‐DPPE) (see Figure S11 in the Supporting Information). The difference in the fluorophores' nature and performance was accounted for by comparing emission spectra of multilamellar vesicles (MLVs) doped with Rh‐DPPE and Rh‐PILs (see Figure S12 in the Supporting Information). From the calibration curve (Figure S11, Supporting Information), we could determine the Rh‐B concentration in the GUV membranes. Accounting for the particle labeling efficiency, we could convert this concentration to a number of PIL particles per membrane area (Figure 3E; see Section S2 in the Supporting Information). These measurements show that the density of particles on the GUV membrane increases with increasing PIL concentration for the PC membranes. However, the highest surface density of ≈1 particle µm^−2^ (found for 10% DOPG membranes with 0.001 × 10^−3^ m PILs) is relatively low considering the small size of the particles. Comparing DOPC and 10% DOPG membranes after incubation with 0.001 × 10^−3^ m PILs, we find that the surface charge of the membrane correlates qualitatively with the binding efficiency of the particles.

The values for the surface coverage have to be considered cautiously. Even though the average vesicle sizes across different compositions follow similar trends (Figure S4, Supporting Information), the history of the individual vesicles examined is not known. In addition, the areas indicated above are projected areas as excess area of the vesicles might have been used to enwrap the particles and fold on itself.

**Figure 4:**
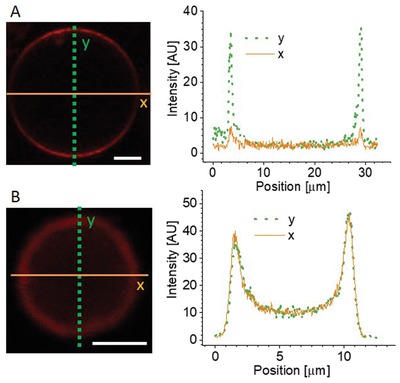
Angular dependence of the fluorescence of Rh‐PILs on GUV membranes. A) Polarization effect exhibited as a strong angular dependence of the intensity of Rh‐B in the PILs along a DOPC vesicle membrane, as emphasized with the line profiles (right) along the respective solid or dashed lines indicated on the image. B) 10% DOPG membranes with no observed polarization effects. The intensity line profiles were generated for a 2 µm wide stripe in the vertical and horizontal directions. Scale bars: 5 µm.

When examining the Rh‐PILs on the GUV membranes, we also noticed differences in intensity as a function of angle, with maximum intensities at the poles of the vesicles. This polarization effect is observed for all vesicles in the DOPC sample at 0.5 × 10^−3^ m PILs, as shown in **Figure** **4**A. For the PILs on the 10% DOPG membranes at 0.001 × 10^−3^ m, we do not observe such polarization effects but rather homogeneous fluorescence over the vesicle contour (see also Figure S13 in the Supporting Information). Note that the polarization effect was observed also for small PC GUVs (data not shown), so size does not govern this phenomenon.

At first sight, this observation seems unimportant as angular dependence of membrane fluorescence has been observed before.^30^ However, the difference between the appearance of DOPC and 10% DOPG vesicles hides an important piece of information about the PIL–membrane interaction and could indicate that there are potentially different interaction mechanisms of the PILs with the membrane, which has a dependence on membrane charge. The angle‐dependent intensity is a result of the dye orientation with respect to the polarization plane of the excitation light, known as the photoselection effect. When fluorophores are illuminated by linearly polarized light, those with transition moments oriented in the same direction as the incident light will be preferentially excited.^31^ This phenomenon has a (cos Θ)^2^ dependence on the angle Θ between the polarization of the incident light and the transition moment of the dye. Such photoselection is commonly observed for Rh‐labeled GUVs^30^ when the dye orientation is aligned with the membrane normal. In the PIL nanoparticles, the dye should have no preferred orientation. Even if it has a specific alignment with the internal structural elements of the PILs, this would be negated by the concentric circular structure of the particles. The photoselection observed in the PC vesicles suggests alignment of the dye with the membrane normal, which would require restructuring of the particle to allow translocation of Rh‐B molecules (which have a lipophilic nature^32^) from the PIL particles to the membrane where the dye aligns with the membrane normal. The stronger interaction between the positively charged polymer and the negatively charged membrane for the 10% DOPG case, as well as the higher particle density, could instead result in rapid engulfment and wrapping of the entire intact particles by the membrane, leaving a fraction of (randomly oriented) Rh‐B in the PILs. Indeed, the significant decrease in vesicle size could be a result of area loss involved in complete engulfment of the particles.

### 2.4 Uptake of PILs Inside GUVs

**Figure 5:**
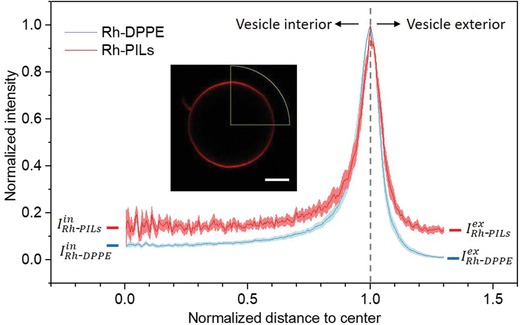
Radial profile of the fluorescence intensity signal averaged over the vesicle azimuthal angle (as shown in the inset; scale bar: 3 μm) and normalized by the maximum value as a function of distance from vesicle center normalized by vesicle size for Rh‐DPPE‐labeled GUVs (blue curve) and DOPC GUVs incubated with 1 × 10^−3^ m Rh‐PILs (red). The intensity values show signal averaged from measurements on 10 GUVs, with the standard deviation shown as the error on the curves (pink and light blue bands).

In addition to measuring the fluorescence intensity from the PILs at the membrane and inspired by an approach developed in ref. 33, we also measured the fluorescence in the GUV interior in the presence of Rh‐PILs. This was done by plotting the radial intensity profiles of confocal images of DOPC vesicles exposed to 1 × 10^−3^ m of Rh‐PILs (because of the poor efficiency of PILs labeling, higher concentration was chosen to ensure sufficiently strong signal from the dye). Similar measurements on 10% DOPG vesicles were not feasible due to the even smaller size as discussed below. The angular averaging negates any effects from polarization of the dye in the membrane, as previously discussed. To avoid interpreting out‐of‐focus membrane intensity as signal from free Rh‐PILs inside the vesicles, we compared these measurements to intensity profiles for PIL‐free GUVs of a similar size but labeled with 0.05 mol% Rh‐DPPE. The out‐of‐focus signal in the interior scales inversely with vesicle size, smaller vesicles having a larger contribution at their center than larger vesicles (see Figure S13 in the Supporting Information). Thus, we measured Rh‐DPPE‐labeled vesicles that were of a comparable size (8–15 µm) to the GUVs with Rh‐PILs. Next, we took the average intensities of profiles as those in **Figure** **5** for the flat region of the curves for values of 0 < *x* < 0.4 on the radii axis, to generate values for interior intensity from Rh‐PILs and out‐of‐focus membrane intensity from Rh‐DPPE‐labeled GUVs ($I_{\mathrm{Rh}‐\mathrm{PILs}}^{\mathrm{in}}$ and $I_{\mathrm{Rh}‐\mathrm{DPPE}}^{\mathrm{in}}$, respectively). The data from the Rh‐DPPE vesicles show clearly the out‐of‐focus contribution as the interior signal is higher than that outside $\left(I_{\mathrm{Rh}‐\mathrm{DPPE}}^{\mathrm{in}}\;>\;\;I_{\mathrm{Rh}‐\mathrm{DPPE}}^{\mathrm{ex}}\right)$. However, the interior signal for the vesicles with Rh‐PILs is even higher $\left(I_{\mathrm{Rh}‐\mathrm{PILs}}^{\mathrm{in}}\;>\;\;I_{\mathrm{Rh}‐\mathrm{DPPE}}^{\mathrm{in}}\right)$ than that for the labeled membranes without Rh‐PILs, suggesting signal from Rh‐PILs in the vesicle interior.

The effective intensity from PILs inside the vesicles can be expressed as the difference $I_{\mathrm{eff}}^{\mathrm{in}}\;=\;\;I_{\mathrm{Rh}‐\mathrm{PILs}}^{\mathrm{in}}\;-\;\;I_{\mathrm{Rh}‐\mathrm{DPPE}}^{\mathrm{in}}$. Similarly, for the exterior of the membrane, $I_{\mathrm{eff}}^{\mathrm{ex}}\;=\;\;I_{\mathrm{Rh}‐\mathrm{PILs}}^{\mathrm{ex}}\;-\;\;I_{\mathrm{Rh}‐\mathrm{DPPE}}^{\mathrm{ex}}$ describes the effective intensity from the PILs outside the vesicles. By comparing these two values, we found that the interior vesicle intensity is 70.3 ± 20.4% of the external intensity value from free Rh‐PILs for 1 × 10^−3^ m PILs with PC vesicles. The internal fluorescent signal strongly indicates that particles have crossed the vesicle membrane. This is likely to happen during the pore formation that occurs when the particles come into contact with the vesicles, as observed during the microfluidic experiments. It also implies that either the pores formed on the membrane are large enough for a PIL particle to permeate or lipid‐wrapped particles detach from the vesicle membrane. Indeed, we do not detect any signal from the membrane dye in the vesicle interior (Figure S15, Supporting Information) ruling out the latter hypothesis and suggesting that bare (lipid‐free) particles permeate through the membrane pores.

### 2.5 PILs Induce Changes in Membrane Properties

**Figure 6:**
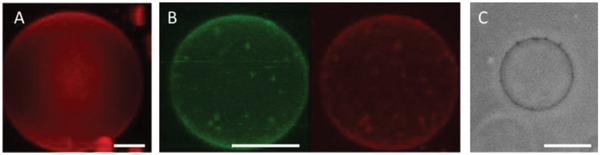
Morphological membrane changes caused by PILs. A) In the absence of PILs, vesicle membranes typically exhibit a smooth surface, as shown in the 3D projection from confocal images of a DOPC vesicle. B) When exposed to 0.1 × 10^−3^ m PILs, these membranes develop lipid clusters and inclusions characterized by the high‐intensity spots (NBD‐PC‐labeled membrane in green, Rh‐PILs in red). C) Similar membrane defects can also be observed in phase contrast. Scale bars: 5 μm.

After the GUV exposure to the PIL particles, we also noticed changes in the vesicle appearance as observed from 3D confocal projections. In the absence of PILs, Rh‐DPPE‐labeled PC vesicles appear smooth and without defects (see **Figure** **6**A). On the contrary, in the presence of PILs, membrane defects are observed (see Figure 6B,C). The defects represent lipid aggregates that are colocalized with increased signal from Rh‐PILs.

Such membrane defects were also observed in phase contrast mode, as shown in Figure 6C. These aggregates of higher optical density could be the result of membrane wrapping around particles and lipid accumulation in their vicinity (or even interior). Given that the size of these membrane defects is well above the average particle size, it could also be possible that upon docking to the vesicle, some of the particles aggregate in clusters, as has been shown both through simulations^34^ and experimentally with micrometer‐sized particles.[[qv: 8d,35]] Regions of dense membrane have also been observed for GUVs exposed to membrane‐active peptides and agents.[[qv: 15,17b,36]] This is a behavior that occurs commonly throughout the literature on cationic membrane‐active molecules. A mechanism to explain this depicts the membrane‐active agents behaving as an intermediate sticky contact between two folded pieces of membrane.^37^

Within the context of membrane properties, we also observed a decrease in lipid fluidity in the presence of PILs. This was assessed using fluorescence recovery after photobleaching (FRAP) on DOPC membranes with and without 0.1 × 10^−3^ m PILs (see the Experimental Section). FRAP is often used to examine changes in membrane fluidity when a fluorophore is placed in different environments, which has direct biological relevance as long‐range lipid diffusion is vital to many membrane processes.^38^

**Figure 7:**
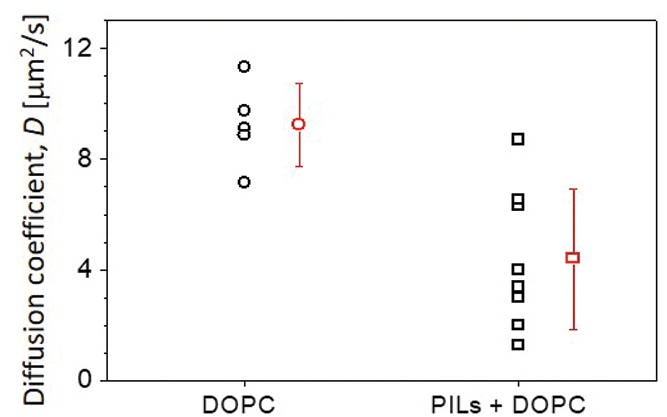
Diffusion in DOPC membranes is slowed down in the presence of PILs. Lipid diffusion was measured using FRAP on DOPC membranes labeled with 0.1 mol% Rh‐DPPE in the absence and presence of 0.1 × 10^−3^ m PILs. Black symbols indicate measurements on individual vesicles; mean and standard deviation are also given (red). Larger scatter was observed for the sample containing PILs.

For DOPC (0.1 mol% Rh‐DPPE‐labeled) GUVs incubated with 0.1 × 10^−3^ m PILs, we observed a decrease in the average lipid diffusion coefficient, from 9.2 ± 1.5 µm^2^ s^−1^ (for vesicles in the absence of PILs) to 4.4 ± 2.5 µm^2^ s^−1^ (see **Figure** **7**). This indicates that the PILs interact with the membrane in such a way as to impinge on the movement of lipids. This could be a sign of particle insertion into the bilayer creating obstacles that the lipids must diffuse around. Alternatively, the particles could condense or tightly bind many lipids simultaneously, such that a significant number of lipids are immobilized on the particle and diffuse slowly with it. The fractions of immobilized lipids during the course of the measurement (see Figure S16 in the Supporting Information) for the control and PIL‐incubated vesicles (0.82 ± 0.03 and 0.74 ± 0.08, respectively) are similar within the error and indicate that the particles reduce the overall diffusion of lipids (or alternatively, the overall membrane state) without lipid immobilization. In addition to the decrease in membrane diffusion, we also observed scatter in the diffusion coefficient values, with individual vesicles in the same sample exhibiting different lipid diffusion. This could be due to differences in properties such as initial membrane tension modulating the PIL–membrane interaction, with some vesicles having more or less excess area for the interactions (resulting in partial wrapping, for example).

## 3 Discussion and Conclusion

**Figure 8:**
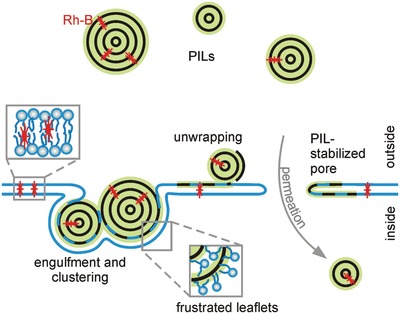
A summary of possible interaction mechanisms between the PIL particles and the GUV membrane. i) PILs become wrapped by the membrane and can cluster; ii) PILs frustrate the lipid bilayer and lipids penetrate the particle and the particle is wrapped by a monolayer of lipids releasing Rh‐B into the membrane hydrophobic core; and iii) this frustrated state or particle engulfment could cause the formation of (nano)pores that allow the entry of PILs in the vesicle interior.

These experimental findings, both individually and in conjunction with each other, lead us to speculate the potential interaction mechanisms of the PILs with the GUVs, a summary of which is shown in **Figure** **8**.

The particles adhere to the membrane, which is revealed by the use of the fluorescently labeled PILs in Figure 3. Some particles might then experience engulfment by the membrane as well as clustering. The visible aggregates on the vesicle colocalized with signal from the membrane itself, as in Figure 6, could be an indication of such a mechanism. Engulfment of particles could also increase membrane tension, by reducing the surface area to volume ratio. The vesicle can then reduce its volume by expelling some of its internal solution through a (macro)pore. Pore formation is inferred via the loss of phase contrast in Figure 1 and directly observed during microfluidic experiments shown in Figure 2. The Rh‐PIL signal in the vesicle interior detected in Figure 5 presumably results from particles entering the GUVs through pores. This, together with the complete exchange of solutions, as shown in Figures 1A,B and 2A, as well as lack of vesicle deformation in electric fields (data not shown) implies longer‐lived, more stable pores (note that for pure lipid membranes pores would reseal quickly due to the high membrane edge tension^23^). Such pores would require stabilization. We speculate that this is achieved by polymer peeling off the PILs. The released polymer intercalates into the bilayer frustrating the leaflets as well as stabilizing the pores as sketched in Figure 8. The particle unwrapping and destabilization is presumably a result of lipids penetrating the PILs' external shell and swelling it similarly to the action of plasticizers that upon embedding between the polymer chains reduce their intermolecular cohesion.^39^ The associated insertion of the polymer chain into the membrane is consistent with the slowdown in the lipid diffusion. The unwrapping of the particle structure is also consistent with the release of the Rh‐B molecules that then incorporate into the membrane (Figure 4) as a result of their lipophilic nature. Moreover, if edge‐active, the unwrapped polymers could stabilize the pores in the membrane. The pores should be large enough to allow the permeation of free PILs as sketched in Figure 8. The transmigration of the free PILs (and in particular the small‐size fraction) could be purely diffusion‐driven whereby the particles could be repelled from the unwrapped polymer lining and stabilizing the pores.

The response of the membranes to PILs can be compared with the pore‐forming action of antimicrobial peptides, which involves the barrel‐stave and toroidal pores.^40^ Briefly, these different mechanisms proceed as follows: for barrel‐stave pores, the helical peptides accumulate on the membrane surface until a threshold concentration where they insert into the membrane and associate to form a stave‐stabilized bundle with a central lumen;^40^ for toroidal pores, the mechanism is similar except that the peptides associate with the lipid head groups and as such the pore is lined by both the peptides and the lipid head groups.^41^ In addition to these pore mechanisms, antimicrobial peptides also interact with membranes via a non‐pore‐forming mechanism, known as the carpet mechanism. In this interaction, the peptides cover the membrane surface until the membrane ruptures or disintegrates in the end.^42^ Such a mechanism could exist for this system as we see an instantaneous decrease in vesicle population upon the addition of particles and occasional macropore formation (Figure 1D).

The PIL‐induced pores appear to be stable for a long time. Presumably, the pore stability is related to the molecular architecture of the lipids and the local curvature generated by them (also referred to as molecular or monolayer spontaneous curvature; see, e.g., ref. 43 for the spontaneous curvature of different lipids). Previous studies have already shown that the lipid type affects pore dynamics^23^ and membrane stability upon poration.^44^ The adsorption of the particles and the potential intercalation of the polymers from the unwrapping PILs (Figure 8) could lead to further changes in the local spontaneous curvature with consequences for the pore stability.

Whether or not the particles purely adhere to the membrane or also start to unravel their structure once they are at the membrane, their presence at the membrane hinders the diffusion of the lipids (Figure 7). The exact mechanism of this slowing of the lipids is not clear, but we propose the following possible explanations. The side chains of the polymers could partially insert into the membrane, creating many small barriers throughout the bilayer. Alternatively, each particle could be binding to many lipids simultaneously, thus creating regions of lipids that cannot diffuse due to the steric hindrance from their neighbors, additionally creating barriers throughout the bilayer. A similar phenomenon has been observed for the lipid diffusion in cell membranes.^45^ The diffusion of lipids in small compartments had a similar diffusion coefficient to comparable synthetic membranes, but the overall diffusion of the cell membrane was significantly lower. The actin‐based membrane skeleton, on which some transmembrane proteins are anchored, created small compartments; the transition of lipids from compartment to compartment was responsible for the overall slowing of the lipids. In our case, the PILs could be creating mobile barriers or platforms.

When considering the potential uses of these particles, it makes sense to draw comparisons with agents that either behave in a similar way or are already employed for the application of interest. As we have observed a lytic action of these PIL particles, with a pronounced contrast between membrane compositions, which can be considered in the context of bacterial membranes, we have compared this action to that of antimicrobial peptides. One important factor to consider is the concentration dependence of these particles. We have found that the PILs produce a biologically relevant interaction when we exceed a monomer concentration of 0.03 × 10^−3^ m (for 40% negatively charged membranes). In comparison, the MBC for the antimicrobial peptide Gomesin (Gm) acting on 1‐palmitoyl‐2‐oleoyl‐sn‐glycero‐3‐phosphatidylcholine (POPC) membranes doped with 25% phosphatidylglycerol (PG) is 0.2 × 10^−6^ m,^16^ two orders of magnitude lower than that of the PILs. This might suggest that the concentration of PILs required to have a significant biological impact is too high and thus cannot compete with such peptides as candidates for antimicrobial agents. However, when one considers that the PIL particles are formed from many repeating monomer units, the effective particle number density is actually several orders of magnitude lower than that for Gm. For the critical concentrations of 0.03 × 10^−3^ m (PILs) and 0.2 × 10^−6^ m (Gm), the number densities are 3.65 × 10^2^ PIL pL^−1^ and 2.73 × 10^11^ Gm pL^−1^, respectively. This implies that many more individual peptides are required to work on the membrane to induce the same response as with the individual PIL particles.

We have also observed events such as stabilized pores and dye polarization that suggests transfer of material from the PILs to the membrane. Such a phenomenon could be utilized for release of active molecules at a target site, for example, localized doses of a drug when the particles bind to a membrane of a specific composition.

Finally, PIL particles as such would open an unexpected way for antibacterial and antifungal coatings. If these films at the same time give a long‐term protection against surface bacterial growth, the standard armament with low molecular weight (and thereby leaching) additives could be avoided.

## 4 Experimental Section

*Materials*: DOPC, DOPG, DOTAP, Rh‐DPPE, and 1‐oleoyl‐2‐[12‐[(7‐nitro‐2‐1,3‐benzoxadiazol‐4‐yl)amino]dodecanoyl]‐*sn*‐glycero‐3‐phosphocholine (NBD‐PC) were acquired from Avanti Polar Lipids (Alabaster, AL). Rhodamine B (>95%), 1‐vinylimidazle (99%), and 1‐bromotetradecane (97%) were purchased from Sigma‐Aldrich and used as received. Indium tin oxide (ITO)‐coated glasses (ITO film thickness < 100 nm, resistance 50 Ω) were obtained from Praezisions Glas & Optik (Iserlohn, Germany). Glucose, sucrose, and BSA were all obtained from Sigma‐Aldrich (Darmstadt, Germany). Low melting temperature agarose was obtained from Fisher Scientific (Waltham, MA). All chemicals were used without further purification.

*Vesicle Preparation*: GUVs were prepared via the established electroformation protocol.^46^ Lipid solutions (4 × 10^−3^ m) were prepared in chloroform with varying ratios of DOPC, DOPG, and DOTAP, as indicated throughout the text. Unless explicitly stated in the text, lipid solutions were prepared in the absence of membrane dyes. The lipid solutions (total volume of 16 µL) were spread on two conductive ITO‐coated glasses and dried under vacuum for 2–2.5 h at room temperature. The ITO glasses together with a Teflon spacer were then assembled to form a chamber (volume 2 mL) that was filled with sucrose (0.2 m). The chamber was then connected to a function generator that was used to apply an AC field (1.2 V, 10 Hz) for 1.5 h at room temperature (for the lipid compositions containing dyes, the electroformation was performed in the dark) or at 60 °C for DOPG‐containing GUVs.[[qv: 27b]] The GUVs were then removed from the growth chamber and diluted 1:1 either in an iso‐osmolar glucose solution or with PILs. Osmolarities were adjusted using an osmometer (Osmomat 030, Gonotec, Germany). Vesicles were allowed to equilibrate for 1 h before observation. Agarose (0.2% w/v) prepared in glucose (0.2 m) was also used to immobilize vesicle populations, both for confocal imaging and for counting vesicle populations.^21^

MLVs were prepared by depositing a drop of lipid solution into a glass round‐bottomed test tube and drying, first under a stream of nitrogen and then under vacuum for 2 h. The lipid film was then rehydrated in water and alternately vortexed and sonicated.

*Particle Preparation and Characterization*: Nonlabeled PIL nanoparticles were prepared as previously reported.^9^ The chemical structure of the PIL is shown in Figure S2 in the Supporting Information. The nanoparticle hydrodynamic size and surface charge were measured using a Zetasizer Nano ZS (Malvern Panalytical, UK). Rh‐PILs were prepared by mixing rhodamine B with ionic liquid monomers prior to polymerization, following the same procedure of the nonlabeled PIL nanoparticles. Dialysis was applied to remove residual rhodamine B after polymerization. The resulting Rh‐PIL concentration was determined by first drying a sample and performing thermogravitational measurements using a thermo‐microbalance TG 209 F1 Libra (Netzsch, Selb, Germany), of which the mass was averaged from four measurements. A platinum crucible was used for the measurement of the dispersion (100 mg) in a nitrogen flow (20 mL min^−1^) and a purge flow (20 mL min^−1^) with a heating rate of 2.5 K. For statistical considerations, a solution of known concentration of polyethylene glycol (10 kDa) was measured in the same way. The stock solution of Rh‐PILs (4.2 wt%) was diluted to the different concentrations used in this manuscript. This concentration value was used to determine the density of a particle configuration (using a density oscillation tube DMA 5000M, Anton Paar, Graz, Austria), which was found to be 1.137 ± 0.003 g mL^−1^. Measuring the fluorescent absorbance of a dried sample resuspended in ethanol, the concentration of rhodamine B in the sample was found to be 0.00091 mg mL^−1^.^31^ By determining the average particle size from transmission electron microscopy images, the average particle mass and therefore the average number of dye molecules per particle can be determined.

*Imaging and Image Analysis*: Phase contrast imaging was performed on an Axio Observer D1 (Zeiss, Germany) microscope, equipped with a Ph2 20 × (NA 0.5) objective and an ORCA R2 CCD camera (Hamamatsu, Japan). Populations were counted manually from five randomly selected regions within each sample and GUV size was measured in ImageJ by fitting circles to the vesicles. Confocal imaging was performed on a Leica confocal SP8 setup (Mannheim, Germany). Rhodamine B and Rh‐DPPE were both excited with a 561 nm laser and NBD‐PC was excited with the 476 nm line of an argon laser. The fluorescence signals for the rhodamine dyes were collected in the range of 570–700 nm, which was adjusted to 620–700 nm in the presence of NBD‐PC to account for crosstalk. The fluorescence signal of NBD‐PC was collected between 483 and 515 nm. The images were collected with 40 × (0.75 NA) dry or 63 × (1.2 NA) water immersion objectives and 1 Airy unit. Image intensity quantification was performed either in the Leica software (Leica Application Suite) using an intensity line profile or by measuring the average membrane intensity within a user‐defined region of interest (ROI); a radial intensity distribution plug‐in in ImageJ was also used to measure intensity as a function of radius (radially averaging accounted for dye polarization effects).

*Microfluidic Preparation and Operation*: The polydimethylsiloxane (PDMS) microfluidic device was fabricated as previously described.^47^ Briefly, PDMS oligomer and curing agent were mixed at a ratio of 10:1 and poured onto the silicon wafer master (feature height: 40 µm) to a final thickness of 5 mm and then cured at 80 °C for 3 h. After cutting to size, 1.5 mm holes were punched using a 1.5 mm Biopsy puncher (Miltex, Plainsboro, NJ). The device was completed and the microfluidic channels sealed by bonding 170 ± 10 µm glass coverslips to the lower side using air plasma (1 min, 0.5 mbar; PDC‐002, Harrick Plasma, Ithaca, NY) and then left at 60 °C for 30 min.

For operation, the device was first filled with BSA (20 mg mL^−1^) dissolved in glucose (0.2 m) (a solution that has first been filtered using 0.45 µm pores) using centrifugation (900 × *g*, 10 min). This creates a protein coating on the walls of the device to minimize vesicle sticking, while the centrifugation ensures a bubble‐free environment. GUVs, PILs, and other solutions were delivered to the device through a reservoir, with a syringe pump (neMESYS, CETONI, Korbussen, Germany) connected to the device at the other end (from the reservoir) and operating in withdrawal mode. The BSA was removed from the device by flushing through with glucose (0.2 m, 20 µL min^−1^, 10 min). The GUVs were loaded into the device in their growth solution of sucrose (10 µL min^−1^, 15 min), after which glucose was added (5 µL min^−1^, 5 min) to induce phase contrast. The PILs were introduced at a reduced flow rate (2 µL min^−1^) and images and time lapses were obtained using the Axio Observer D1.

*Fluorescence Recovery After Photobleaching*: FRAP measurements were performed on membranes labeled with Rh‐DPPE (0.1 mol%). Images were recorded at 1400 Hz with a pinhole size of 1 Airy unit in bidirectional mode and with an image size of 296 × 296 pixels, using the 561 nm laser. Before bleaching, 10 frames at attenuated laser intensity (below 5%) were recorded. The photobleaching was performed for 200 ms (three frames) at 100% laser intensity using a circular ROI of nominal radius *r*_n_ = 1 µm (see Figure S15 and Section S3 in the Supporting Information). The postbleach recovery images were then recorded at the initial attenuated laser intensity for several seconds. The photobleaching was always conducted on the upper or lower vesicle surface. For details on the data analysis, see Section S3 in the Supporting Information.

## Conflict of Interest

The authors declare no conflict of interest.

## Supporting information

Supplementary

Supplementary
