# Supplementary material for: Poly(Ionic Liquid) Nanoparticles Selectively Disrupt Biomembranes
Source: Adv Sci (Weinh). 2018 Dec 17;6(4):1801602. doi: 10.1002/advs.201801602 (PMC6382306; doi:10.1002/advs.201801602)
Supplement: Supplementary file 1 — Supplementary [file ADVS-6-1801602-s002.pdf]

Copyright WILEY-VCH Verlag GmbH & Co. KGaA, 69469 Weinheim, Germany, 2018.

ADVANCED SCIENCE
Open Access

Supporting Information

for *Adv. Sci.,* DOI: 10.1002/advs.201801602

Poly(Ionic Liquid) Nanoparticles Selectively Disrupt Biomembranes

*Eleanor Ewins, Rafael B. Lira, Weiyi Zhang, Jiayin Yuan, Markus Antonietti, Tom Robinson, and Rumiana Dimova**

Poly(ionic liquid) nanoparticles selectively disrupt biomembranes

Eleanor Ewins,† Rafael B. Lira,† Weiyi Zhang, ‡,§ Jiayin Yuan, ‡,§ Markus Antonietti,‡ Tom Robinson,† and Rumiana Dimova*†.

†Departments of Theory & Bio-Systems, and ‡Colloid Chemistry, Max Planck Institute of Colloids and Interfaces, Science Park Golm, 14424 Potsdam, Germany

§ Current address: Department of Materials and Environmental Chemistry, Stockholm University, 106 91 Stockholm, Sweden

*Address correspondence to: dimova@mpikg.mpg.de

Supplementary Information

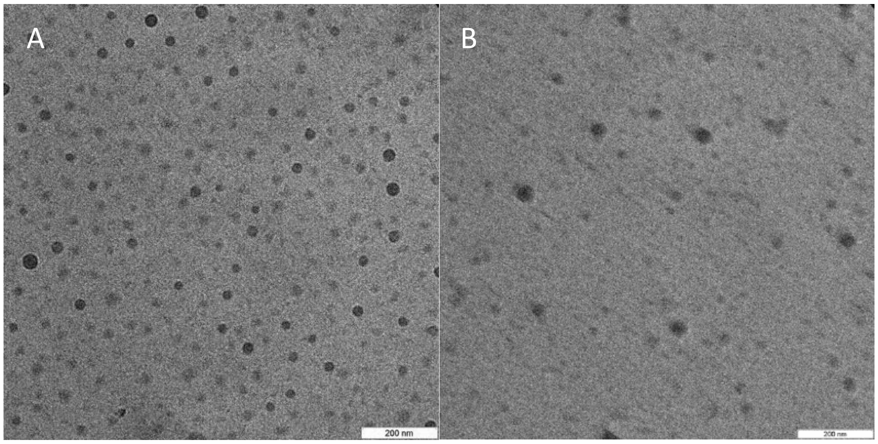

Figure S1. TEM images of PIL samples used for determining average particle size. (A) Unlabeled PILs used for bulk incubation experiments. (B) TEM images of Rh-B PILs. To measure the particle radius, we fitted circles to the particles in ImageJ for 20 random PILs from 3 TEM images. The average diameter of the PILs was found to be 24.0±6.5 nm, and that of the Rh-PILs 37±11 nm. Scale bars: 200 nm.

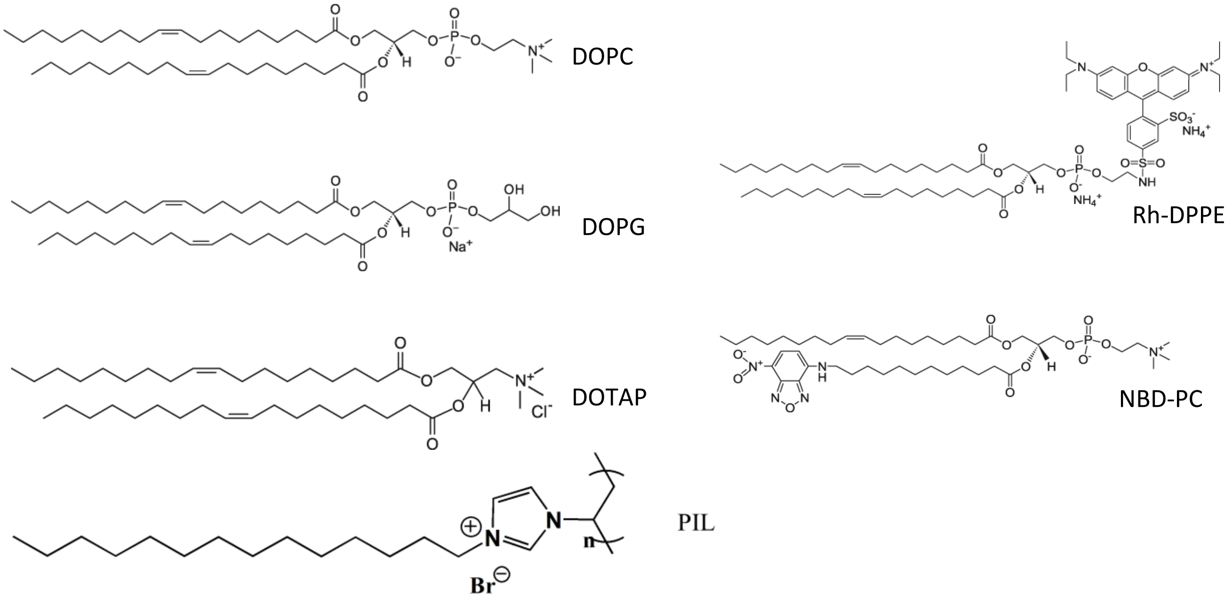

Figure S2. Structures of used lipids, lipid dyes and PIL monomer.

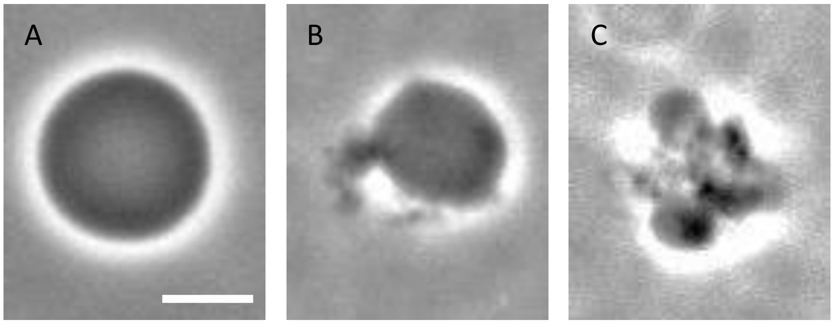

Figure S3. Included and excluded vesicle examples. (A) Typical example of a vesicle included in statistics. (B) and (C) typical examples of vesicles not included in statistics. Scale bar: 5 µm.

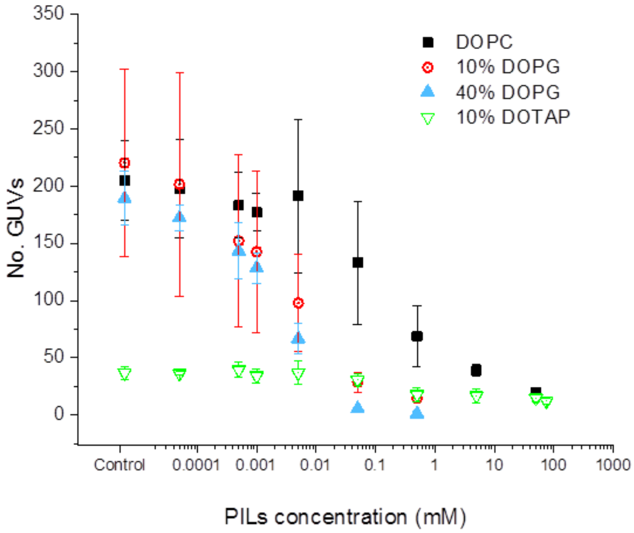

Figure S4. Raw data for the number of surviving vesicles in a population with increasing PILs concentration.

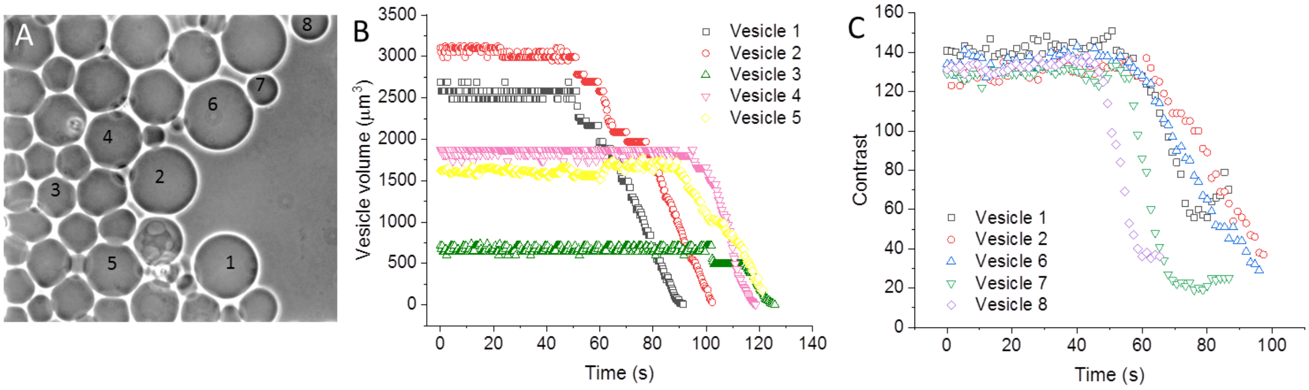

Figure S5. GUVs exposed to PILs in microfludic device and their subsequent changes in volume and contrast over time from microfluidic time lapses. (A) Vesicles trapped in device before exposure to PILs, numbers denote which vesicle is being analyzed within plots in (B) and (C). The PILs solution is introduced from the right. (B) Vesicle volume determined from fitting circle in ImageJ to contour, assuming a spherical GUV shape. The onset of vesicle volume decrease also corresponds to the location in the chamber the GUV is; vesicles situated further from the source of the PILs start decreasing later on. (C) Exchange of sugar solutions assessed from intensity line profile across membrane, where contrast is the difference between the maximum and minimum gray scale values (AU). Note the inflection point at ~ 80 s for vesicle 1, which corresponds to a thickening in vesicle membrane, artificially increasing the contrast across the membrane.

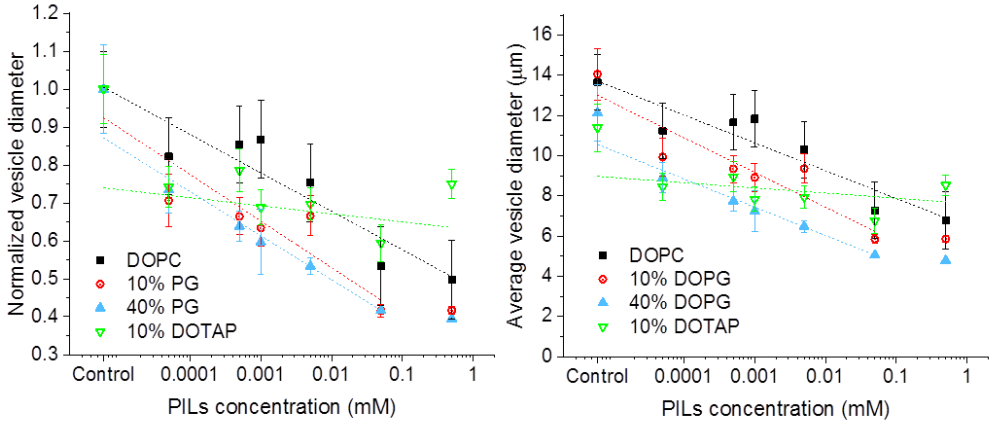

Figure S6. Changes in average vesicle size for all lipid compositions, normalized (left) and raw (right) data. The error bars represent standard deviation from three independent measurements. The linear fits show the trends.

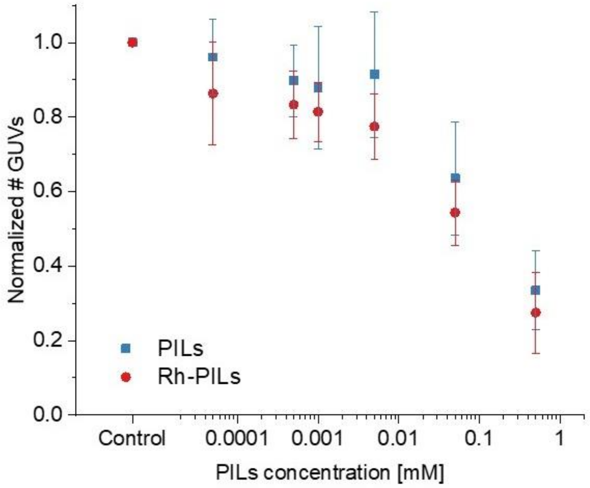

Figure S7. Comparison between change in vesicle population for DOPC membranes with labeled Rh-PILs and non-labeled PILs.

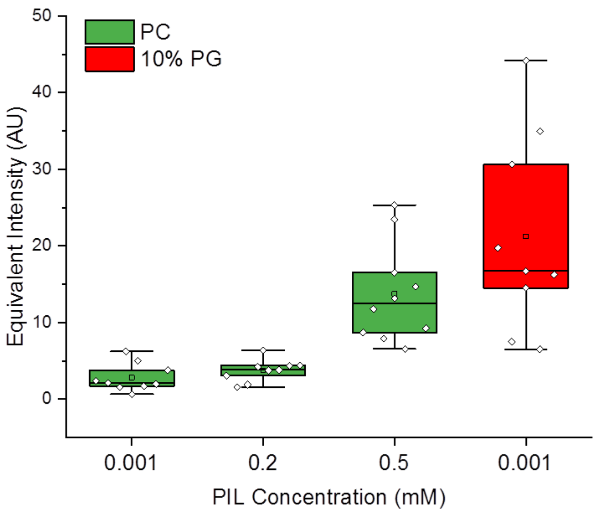

Figure S8. Intensity measurements from Rh-PILs on DOPC membranes, showing the spread of the data for the measured intensity values.

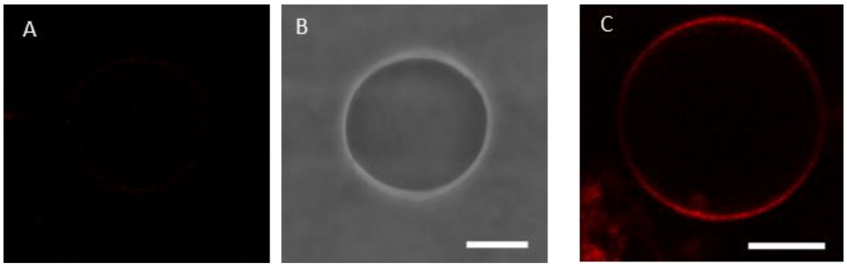

Figure S9. Comparison between non-labeled membranes without (A, B) and with Rh-PILs (C). (A) Confocal cross section of non-labeled vesicle. (B) Phase contrast image of same vesicle as in (A). (C) Confocal cross-section of non-labeled DOPC GUV incubated with 0.5 mM Rh-PILs. This illustrates that fluorescent signal on membrane in contact with Rh-PILs is not a result of background signal or fluorescent contamination of vesicle sample. Scale bars: 5 µm.

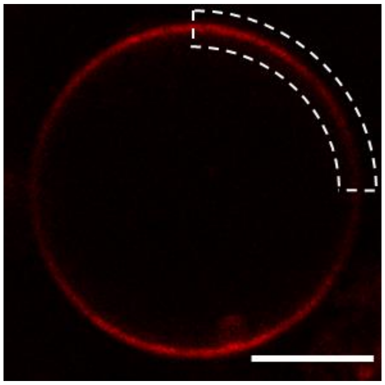

Figure S10. Example membrane intensity measurement used to produce the calibration curve (Fig. S9) and to measure intensity of PILs on membranes. The polarization effect is visible from the angular change in the intensity. An area including a quarter of the vesicle is selected (as indicated with the dashed line) to average out this effect. From the selected region, the average pixel intensity is measured with LAS X Leica confocal microscopy software. Scale bar: 5 µm.

Section S1. Calculation of the labeling efficiency of the Rh-PILs

In order to calculate the area occupied by a single PILs particle on the membrane (via the Rhodamine B, Rh-B, fluorescence), the labeling efficiency was first determined, i.e. the number of Rh-B molecules per individual PILs particle. The diameter (d) of the particles from TEM images (Fig. S1) was found to be 37 ± 11 nm, which gave a volume (V) of a single particle as 2.65×10^{-17} ml. The mass of a single particle (m_{PILs}) was calculated using, $m_{PILs} = \rho \times V$, (the particle density is ρ =1.137 ± 0.003 g/ml, see Materials and Methods in the main text) and found to be 3.01×10^{-14} mg. The number density of PILs (n_{PILs}) was then calculated using,

$$n_{PILs} = \frac{[PILs]}{m_{PILs}} = 4.97 \times 10^{14} \text{ particles/ml}$$

where $[PILs]$ is the total PILs concentration given as 14.96 mg/ml. The concentration of Rh-B measured by absorbance was $[RhB] = 0.00091$ mg/ml = 1.8998×10^{-6} mol/L, which gives the number density of Rh-B (n_{RhB}) as

$$n_{RhB} = [RhB] \times N_A = 1.14 \times 10^{15} \text{ molecules/ml}$$

Therefore, the number of Rh-B molecules to PILs particles was calculated using,

$$\frac{n_{RhB}}{n_{PILs}} = \frac{1.14\times10^{15}}{4.97\times10^{14}} = 2.3 \text{ Rh-B/particle} \qquad \text{(S1)}$$

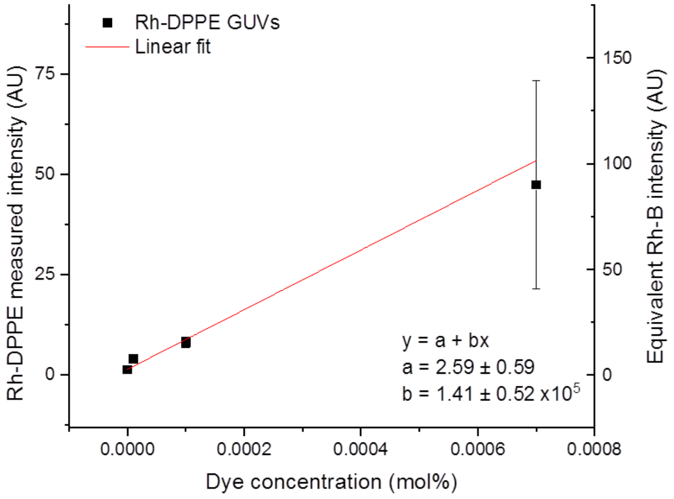

Figure S11. Calibration curve of the measured fluorescence intensity of GUVs (left axis) as a function of Lissamine Rhodamine-B DPPE (Rh-DPPE) concentration in their membrane. The Rh-DPPE concentration was varied from 0 to 0.004 mol%. The GUV fluorescence intensities were then converted to equivalent Rh-B intensities (right axis) based on the fluorescence correction factor between these two dyes as assessed in Fig. S12. The linear fit (red line) of the equivalent Rh-B intensities can be subsequently used to determine the concentration of Rh-B in the membrane from the fluorescence intensities of GUVs in the presence of Rh-PILs as measured in the main text.

Section S2. Calculation of area per PIL on the GUV membrane

The concentration of Rh-B in PILs on membrane, $[RhB_{membrane}]$, could be determined using the fit from the calibration curve in Fig. S9 and the intensity of Rh-B measured from the GUVs,

$$[RhB_{membrane}] = \frac{Intensity - 2.59}{1.41} \text{ mol\%}$$

This allows the calculation of of PILs concentration on membrane using the Rh-B concentration and the ratio of Rh-B to PILs number densities (Eq. S1)

$$[PILs_{membrane}] = \frac{[RhB_{membrane}]}{\frac{n_{RhB}}{n_{PILs}}} \text{ mol\%}$$

Finally, the area per PIL was found using the area a single DOPC lipid headgroup occupies (0.7 nm^2) and the PILs concentration on the membrane,

$$\text{Area per PILs} = \frac{100\ (lipids)}{[PILs_{membrane}](mol\%)} \times 0.7 \text{ nm}^2$$

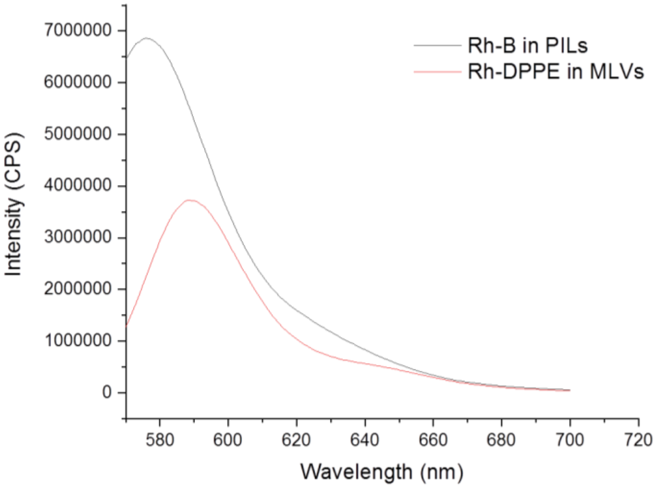

Figure S12. Emission spectra for 9.1 × 10^{-4} mg/ml of Rh-DPPE in MLVs and Rh-B in PILs (this concentration is the measured concentration of Rh-B in the PILs sample after synthesis) used to correct for differences in the fluorophores and their performance. The data was collected with a fluorimeter FluroMax-4 (Horiba, Germany) using a quartz cuvette, which was rinsed (using ethanol and water) and dried between each sample measurement. The excitation wavelength was set to 561 nm and the emission was collected from 570 – 700 nm, matching the confocal laser and detector settings used for imaging. The fluorescence emission of each sample was collected from 570-700 nm for an excitation of 561 nm (slit width 1 nm). The area under each emission curve was calculated and the ratio between the area under the curves for the two samples was determined for three dye concentrations (0.1 × 10^{-5} mg/ml, 4.5 × 10^{-4} mg/ml and 9.1 × 10^{-4} mg/ml). The average ratio between the Rh-B in the PILs and the Rh-DPPE in the MLVs was found 1.905 ± 0.002 and was used to correct for the difference in the performance of the fluorophores in Fig. S9.

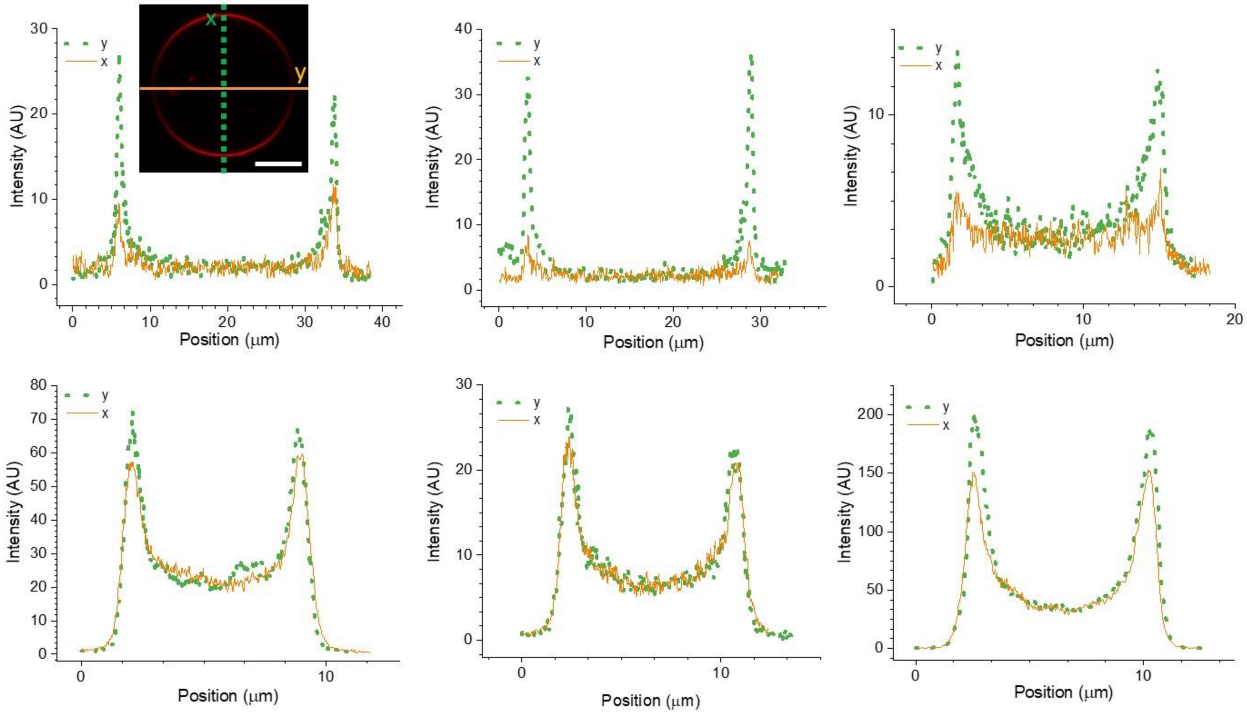

Figure S13. Representative intensity line-profiles of different DOPC vesicles with 0.5 mM Rh-PILs (top panel) and 10% DOPG vesicles with 0.001 mM Rh-PILs (bottom panel) respectively. The inset in the first graph shows an example confocal cross section with the x- and y-lines along which the intensity signal is measured (scale bar 5 µm). The line intensity profiles in the x and y directions of GUVs show that there is notable polarization effect for DOPC membranes and significantly less such for 10% DOPG membranes.

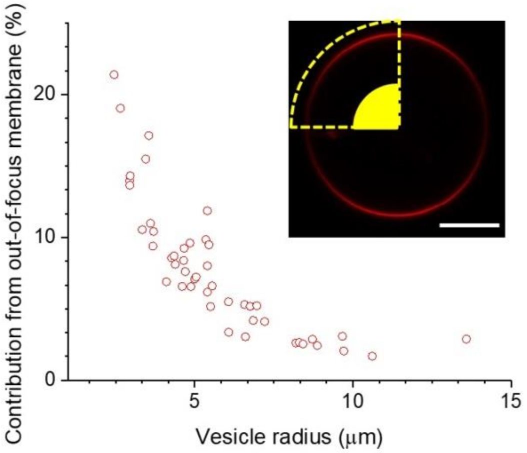

Figure S14. Plot showing membrane interior intensity for the 1st 40% of the distance from the center to the vesicle radius as a function of vesicle size. We measure vesicle intensities using the ImageJ radial angle intensity plugin for GUVs of different sizes. Then, we calculate the average intensity for the central 40% of the vesicle (counting from the center outwards) and determine what percentage this region is of the maximum membrane intensity value (segment in the inset is for illustrative purposes only showing these 40%). We then plot these values as a function of vesicle size, which clearly demonstrates the increased contribution of out-of-focus fluorescence for smaller vesicles. Scale bar on confocal inset 5 μm.

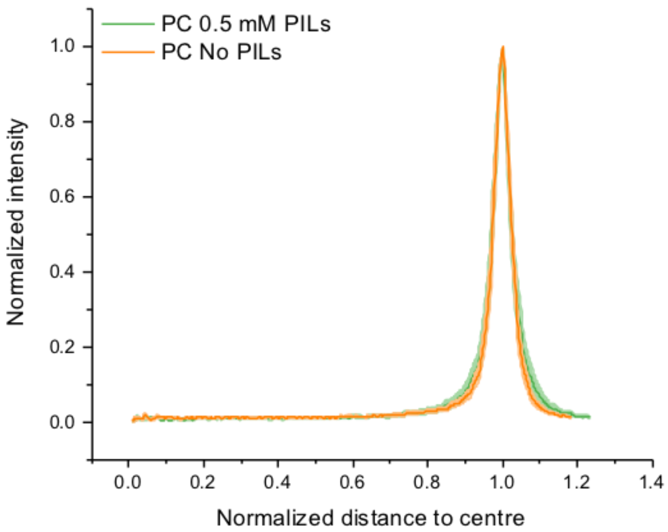

Figure S15. Radial profile of the fluorescence intensity signal averaged over the vesicle azimuthal angle and normalized by the maximum value as a function of distance from vesicle center normalized by vesicle size for NBD-PE labeled DOPC GUVs with (green curve) and without (orange curve) 0.5 mM PILs. The intensity values show signal averaged from measurements on 10 GUVs, with the standard deviation shown as the error on the curves (orange and light-green bands).

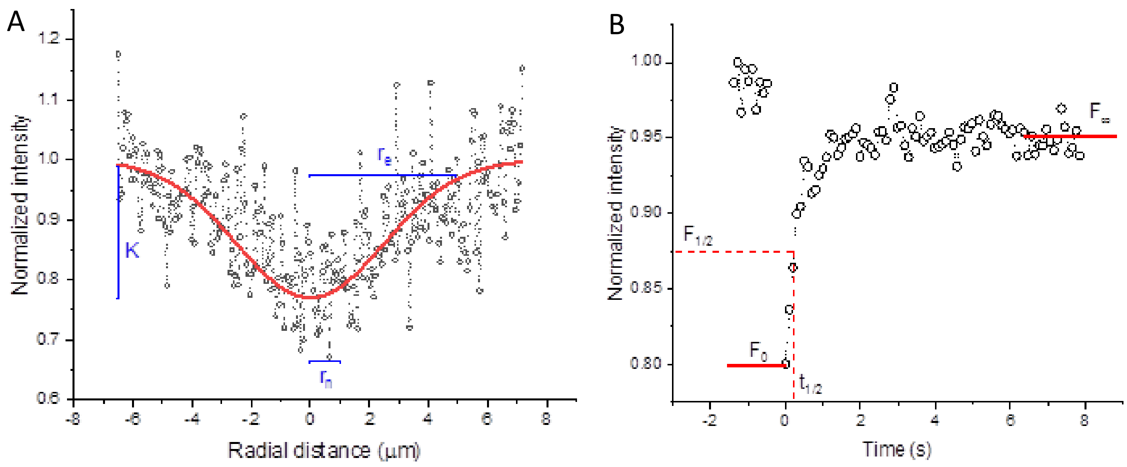

Figure S16. Determination of values used for calculating lipid diffusion coefficients. (A) Intensity line profile of the first frame post-bleaching. The red line is a fit of the data using the equation $(x) = 1 - K\exp\left(\frac{-2x^2}{r_e^2}\right)$ and from this the value of r_e for each vesicle is obtained. The value of r_e used for the calculation of each diffusion coefficient is an average of the r_e values obtained for vesicles in the same sample. In the diagram, the parameters K and r_n are also labeled in blue. (B) Example FRAP recovery for the same GUV as shown in (A), formed from DOPC and immobilized in 0.5% w/v agarose. F_0 is the intensity of the ROI in the first frame post-bleaching, F_∞ is the intensity after the membrane has recovered and $t_{1/2}$ is the time taken for the intensity to recover by half. The immobile fraction is reflected by the difference between the pre-bleach signal to the recovered one, rescaled by the latter.

Section S3. Analysis of the FRAP data

There are several methods reported in the literature [1] with which to extract the lipid diffusion coefficient D from the FRAP recovery curves. Here, we chose to analyze the data using a simplified equation which also accounts for the molecular diffusion during photobleaching[2] which subsequently reduces the error on our values. We obtained the diffusion coefficient D from the following relationship:

$$D = \frac{r_e^2 + r_n^2}{8t_{1/2}}$$

in which r_e and r_n are the effective and nominal bleaching radii respectively, and $t_{1/2}$ is the half-life of the recovery of the fluorescence after bleaching (defined as the time taken for $F_{1/2} = (F_0 + F_\infty)/2$, where F_0 is the fluorescence intensity of the ROI in the first image post-bleach and F_∞ is the fluorescence intensity in this same region after full recovery; see Fig.). The effective bleaching radius, r_e, was obtained by plotting a fluorescence intensity line profile, f(x), through the center of the bleaching region in first frame after photobleaching and fitting the data to this expression:

$$f(x) = 1 - K\exp\left(\frac{-2x^2}{r_e^2}\right)$$

In this equation, K is the bleaching depth and x the radial position. In our experiments, values of r_e = 3.50 μm and r_e = 3.35 μm were used for the control and particle containing samples respectively. An example determination of these values can be found in Fig. S14, along with a typical recovery curve. Also from the recovery curve, it is possible calculate the mobile fraction (M_f) as

$$M_f = \frac{F_\infty - F_0}{F_i - F_0}$$

where F_i is the intensity before photobleaching.

Movie S1: Time lapse showing the interactions for 0.1 mM PILs with 10% PG GUVs using a microfluidic device to introduce nanoparticles, recorded with a frame rate of 13 frames per second. The microfluidic device holds a fixed population of GUVs (that we had grown in sucrose) in place after we had flushed an osmotically matched glucose solution through the chamber. As the the PILs are introduced from the right, we can observe several different morphological changes, such as membrane shrinkage, loss of contrast and bursting. A synopsis of these changes is in Fig. 2 in the main text. The total length of the recording in real time is 2 min and 29 sec.

References

[1] a) D.M. Soumpasis, *Biophys. J.*, 41 (1983) 95-97; b) D. Axelrod, D.E. Koppel, J. Schlessinger, E. Elson, W.W. Webb, *Biophys. J.*, 16 (1976) 1055-1069; c) M. Kang, C.A. Day, K. Drake, A.K. Kenworthy, E. DiBenedetto, *Biophys. J.*, 97 (2009) 1501-1511.
[2] M. Kang, C.A. Day, A.K. Kenworthy, E. DiBenedetto, *Traffic*, 13 (2012) 1589-1600.
